# Macrophage Sub-Populations and the Lipoxin A_4_ Receptor Implicate Active Inflammation during Equine Tendon Repair

**DOI:** 10.1371/journal.pone.0032333

**Published:** 2012-02-22

**Authors:** Stephanie Georgina Dakin, Dirk Werling, Andrew Hibbert, Dilkush Robert Ephrem Abayasekara, Natalie Jayne Young, Roger Kenneth Whealands Smith, Jayesh Dudhia

**Affiliations:** 1 Department of Veterinary Clinical Sciences, Royal Veterinary College, University of London, Hatfield, United Kingdom; 2 Department of Pathology and Infectious Diseases, Royal Veterinary College, University of London, Hatfield, United Kingdom; 3 Department of Veterinary Basic Sciences, Royal Veterinary College, London, UnitedKingdom; 4 Department of Biotherapeutics, National Institute for Biological Standards and Control, South Mimms, United Kingdom; Université de Montréal, Canada

## Abstract

Macrophages (Mϕ) orchestrate inflammatory and reparatory processes in injured connective tissues but their role during different phases of tendon healing is not known. We investigated the contribution of different Mϕ subsets in an equine model of naturally occurring tendon injury. Post mortem tissues were harvested from normal (uninjured), sub-acute (3–6 weeks post injury) and chronically injured (>3 months post injury) superficial digital flexor tendons. To determine if inflammation was present in injured tendons, Mϕ sub-populations were quantified based on surface antigen expression of CD172a (pan Mϕ), CD14^high^CD206^low^ (pro-inflammatory M1Mϕ), and CD206^high^ (anti-inflammatory M2Mϕ) to assess potential polarised phenotypes. In addition, the Lipoxin A_4_ receptor (FPR2/ALX) was used as marker for resolving inflammation. Normal tendons were negative for both Mϕ and FPR2/ALX. In contrast, M1Mϕ predominated in sub-acute injury, whereas a potential phenotype-switch to M2Mϕ polarity was seen in chronic injury. Furthermore, FPR2/ALX expression by tenocytes was significantly upregulated in sub-acute but not chronic injury. Expression of the FPR2/ALX ligand Annexin A1 was also significantly increased in sub-acute and chronic injuries in contrast to low level expression in normal tendons. The combination of reduced FPR2/ALX expression and persistence of the M2Mϕ phenotype in chronic injury suggests a potential mechanism for incomplete resolution of inflammation after tendon injury. To investigate the effect of pro-inflammatory mediators on lipoxin A_4_ (LXA_4_) production and FPR2/ALX expression *in vitro*, normal tendon explants were stimulated with interleukin-1 beta and prostaglandin E_2_. Stimulation with either mediator induced LXA_4_ release and maximal upregulation of FPR2/ALX expression after 72 hours. Taken together, our data suggests that although tenocytes are capable of mounting a protective mechanism to counteract inflammatory stimuli, this appears to be of insufficient duration and magnitude in natural tendon injury, which may potentiate chronic inflammation and fibrotic repair, as indicated by the presence of M2Mϕ.

## Introduction

Pathologic changes in tendons due to repetitive use are a significant cause of morbidity in humans, with Achilles tendon injury rates of up to 56% amongst elite athletes [1], [2]. The corresponding tensile region of the equine superficial digital flexor tendon (SDFT) is similarly susceptible to exercise induced tendinopathy [3], which may be exacerbated by sudden overstrain injury. The importance of inflammatory or degenerative processes contributing to tendon injuries is subject to debate. In humans, tendinopathy is described as a result of a primarily degenerative condition, as inflammatory cells are rarely observed in histological studies of over-strained or ruptured human tendons [4], [5], [6], [7], [8]. However, this may be attributable to factors such as the timing at which human patients present themselves for examination, which is usually at the later stages of injury (or with recurrent injury), and the availability of tissues for examining at different times after injury, precluding study of the early phases of tendon repair. In naturally occurring equine tendinopathy, there is an initial transient phase (days) of clinically evident inflammation (heat, pain, swelling), which rapidly subsides [9]. The sub-acute phase (weeks) is characterised by the resolution of symptomatic inflammation and the onset of fibroplastic repair. In chronic injury the repair continues for many months but in both man and horse the normal architecture, composition and function of tendon are never restored after injury [6], [10], [11].

Macrophages (Mϕ) are an important immune cell type involved in both inflammatory and repair-processes. Indeed, Mϕ are present in chronically injured samples of torn human supraspinatus tendon [12]. Furthermore, rodent models of surgically induced tendon injury have been used to evaluate the presence of inflammatory cells by immunohistochemistry [13], [14]. In these experiments, injury elicited a sequential pattern of inflammatory cell infiltration, consisting of a rapid and transient accumulation of neutrophils, followed by an increase in Mϕ infiltration between 1 and 28 days post injury in a rat model of Achilles tendon injury [13]. Similarly, Wong and co-workers documented temporal changes in inflammatory cell subsets in a murine immobilised surgical adhesion model of injury, reporting peak neutrophil and Mϕ accumulation 1–5 days and 21 days post surgery respectively [14]. The requirement of Mϕ for adult tissue repair is supported by the use of wound healing studies in murine Mϕ-knockout models, with impaired healing responses observed in Mϕ deplete wounds [15], [16], [17]. Although crucial for healing, Mϕ initially fulfil a different function by secreting pro-inflammatory agents in response to tissue damage, including interleukin-1-beta (IL-1β), Tumour Necrosis Factor alpha (TNFα), bioactive prostaglandins, reactive oxygen intermediates and many proteases [18]. These factors have been shown to act as important initiators of the tendinopathic cascade [19], [20], [21], [22], which may drive matrix metalloproteinase (MMP) mediated catabolism of tendon extracellular matrix [21], [22], [23].

In order to identify Mϕ in normal and injured tendons we selected CD172a, as this antigen has been used to successfully identify Mϕ in ovine [24] and equine [25] tissues. The unique plasticity in function of Mϕ can in part be attributed to different subsets/phenotypes. Mϕ have the capacity to exist in at least two functionally distinct phenotypic states, differing in their cell surface receptor expression, effector function and cytokine production [26], [27], [28], [29]. M1 polarised (classically activated) Mϕ appear to show a pro-inflammatory response pattern, whilst M2 (alternatively activated) Mϕ dampen inflammatory responses by producing immunosuppressive cytokines such as IL-1 receptor antagonist (IL-1Ra), IL-10, IL-4 and IL-13 [28]. Expression of both M1 and M2 Mϕ markers is reported in circulating peripheral blood mononuclear cells [30], [31]. M1Mϕ surface antigens include CD14 [32], [33], [34], CD16, CD32, CD64, CD80 and CD86 [28], but not the markers of M2Mϕ polarisation which include the mannose receptor (MR) CD206 and CD163 [28], [30], [34]. Therefore, M1Mϕexpress CD14, but not CD206 i.e. (CD14^high^CD206^low^), whereas M2Mϕ express CD206 but not necessarily CD14 (CD14^low^CD206^high^) and so this combination of surface antigens permits identification of the predominating Mϕ phenotype. The temporal changes in Mϕ phenotype influence synthesis of the extracellular matrix of the injured tissue throughout the different phases of wound healing [16], [35]. Furthermore a correlation between the degree of inflammation and development of subsequent fibrosis has been reported, which is thought to be a Mϕ mediated progression [36].

Despite evidence suggesting that excessive inflammation causes damage to connective tissues by initiating the fibrotic repair that ensues, total inflammatory blockade may not necessarily be beneficial for wound healing processes [37], [38]. An intriguing dimension to the physiology of inflammation is the identification of specialised pro-resolving mediators (SPM) encompassing lipoxins such as lipoxin A_4_ (LXA_4_) [39] acting upon FPR2/ALX (previously termed FPRL1 or ALXR) [40] and more recently, the resolvins, protectins and maresins [41], [42]. FPR2/ALX is expressed by monocytes and Mϕ [43] and is central to controlling the duration and magnitude of the inflammatory response by modulating leukocyte adherence and chemotaxis, and in so doing provides ‘endogenous stop signals’ for inflammation [44]. Although stereoselective for LXA_4_, FPR2/ALX is a multi-recognition receptor [45], to which the anti-inflammatory protein Annexin A1 (ANXA1) also binds [46], [47], [48]. Annexin A1 is also implicated in the resolution of inflammation, with additional important roles in phagocytosis and apoptosis [49], [50]. These resolving pathways are programmed responses activated during inflammation, demonstrating critical roles in the switching of lipid mediators from the prostanoid to the lipoxin axis in order to promote resolution of inflammation and the return of tissues to their normal homeostatic state [39], [44], [51], [52], [53]. Despite the anticipated importance of Mϕ, SPMs and Annexin A1 in healing tendons, there is limited information documenting their presence in the early stages of tendon injury. The presence of SPMs has not been previously assessed in tendons, although studies in other inflamed connective tissues have emphasised their importance in resolving inflammation, including inhibition of neutrophil and Mϕ recruitment and modification of vascular permeability [44].

Based on the similarities between tendon healing in horses and humans [54], [55], studies in naturally occurring equine tendinopathy may be physiologically representative of equivalent injury in humans. For example, aging of the tendon matrix is believed to be an important contributing factor to the development of tendinopathy [56], [57], yet the effects of aging are restricted in rodent models in contrast to those species displaying more evident aging phenotypes such as horse and man [58], [59], [60]. Moreover, the strain experienced during peak loading of the elastic energy storing tendons in equine and human Achilles tendon *in vivo* far exceed those sustained by rodent models [54], [61], [62]. Furthermore, re-injury of chronically diseased tendons is common during rehabilitation of patients with tendinopathy, a scenario which is difficult to recapitulate in murine models of tendon injury. Thus analysis of injured equine tendon, particularly during the early phase of injury, presents a more appropriate and readily attainable source than the human counterpart, and analysis of naturally diseased tissues may be more representative than induced models of tendon injury [63], [64]. Our analysis provides evidence for potential changes in Mϕ sub-populations, FPR2/ALX and Annexin A1 expression during stages of flexor tendon healing compared to normal (uninjured) tendons. Upregulation of FPR2/ALX expression by tenocytes in sub-acute injury *in vivo* was supported by *in vitro* experiments assessing the effect of pro-inflammatory mediators on tendon.

## Results

### Macroscopic and microscopic analysis of injured equine tendons

**Figure 1:**
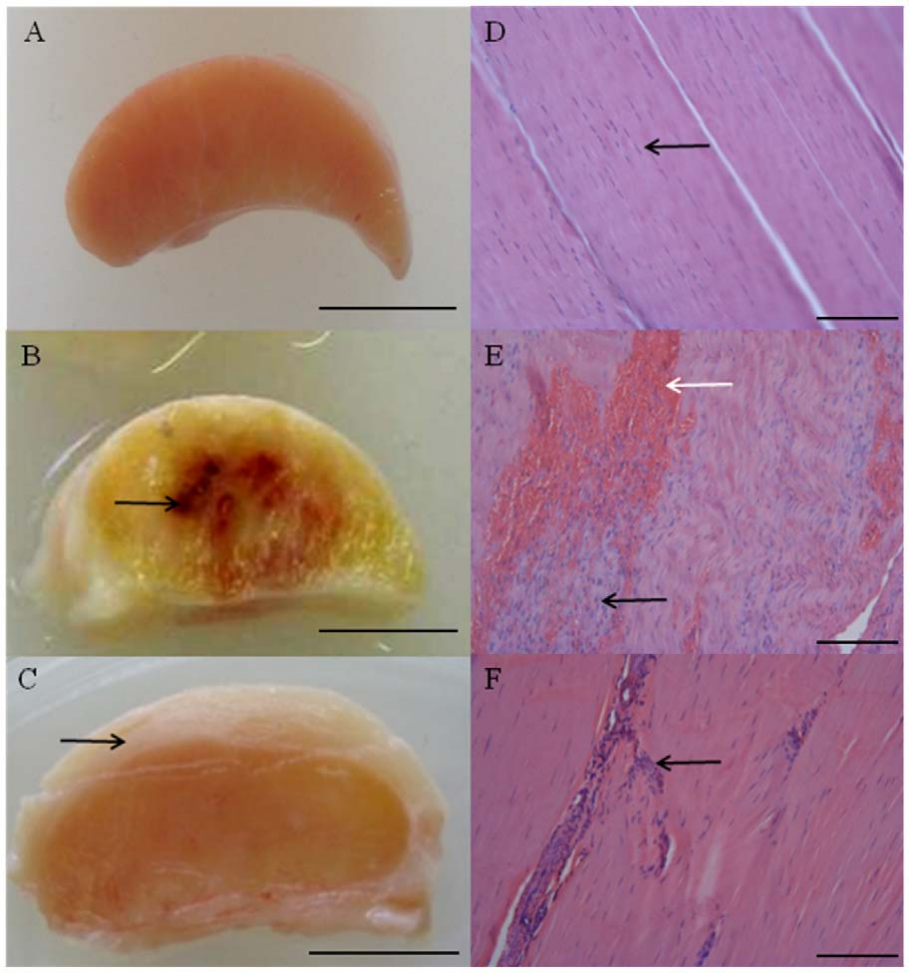
Typical macroscopic appearance of normal and injured equine flexor tendons. (A) Normal superficial digital flexor tendon (SDFT) from a 12 year old horse; (B) sub-acutely injured SDFT 3 weeks post injury from a 4 year old horse, exhibiting a haemorrhagic granular central core (arrow) and (C) chronically injured SDFT >3 months post injury with a thickened fibrosed paratenon (arrow) from a 12 year old horse. Scale bar for macroscopic images = 1 cm. Corresponding longitudinal histology sections stained with Haematoxylin and Eosin: (D) normal SDFT showing regular arrangement of collagen fibrils (arrow); (E) sub-acutely injured SDFT with marked cellular infiltration (black arrow) and haemorrhage (white arrow); (F) chronically injured SDFT with increased cellularity in peri-vascular regions (arrow). Histology scale bar = 12.5 µm.

**Figure 2:**
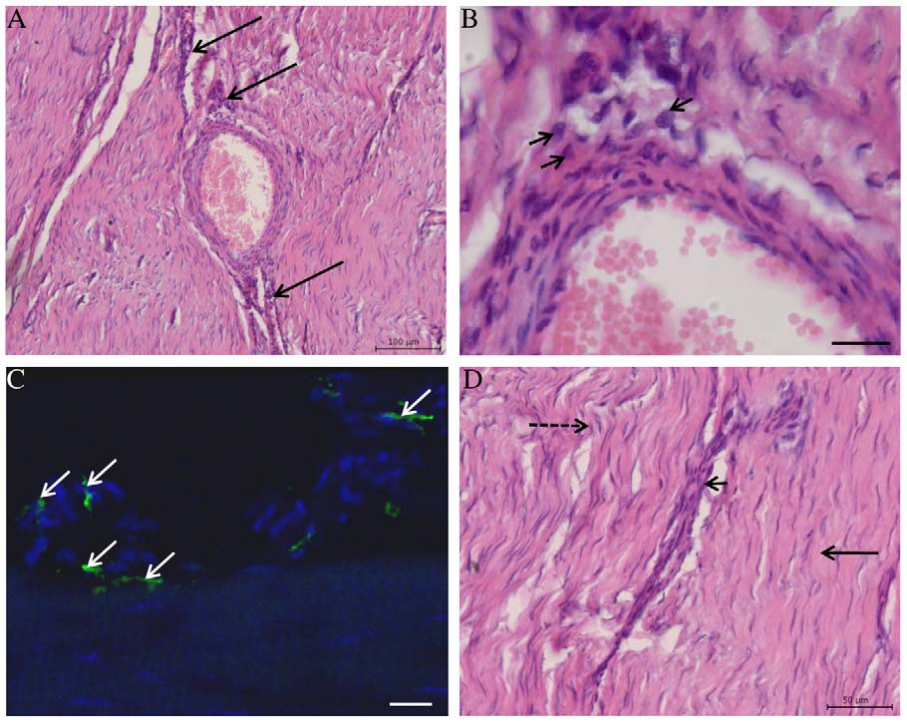
Haematoxylin and Eosin stained longitudinal histology sections of chronic injured SDFT (>3 months post injury) from a 7 year old horse (A, B and D). (A) Reactive fibroplasia with increased cellularity in peri-vascular region (arrows). Scale bar = 100 µm. (B) Higher magnification of (A) showing presence of macrophages (arrowheads) in peri-vascular areas. Scale bar = 20 µm. (C) Corresponding 3-dimensional reconstructed Z stack image of dual antibody labelling for CD14 (red) and CD206 (green). Blue represents Hoechst nuclear counter stain. White arrows show CD14^low^CD206^high^ M2Mϕ located in peri-vascular endotenon regions. Scale bar = 20 µm. (D) Histological appearance of more normal SDFT to the right of the image (straight arrow) in contrast to irregular arrangement of collagen fibrils on the left (dashed arrow). The linear interface between more normal and injured zones of tendon is demarcated by an area of increased cellularity containing macrophages (arrowhead). Scalebar = 50 µm.

Normal SDFT's (Fig. 1A) were smaller in size compared to sub-acutely injured tendons, which exhibited a central core of haemorrhagic granulation tissue and disruption of the fascicle arrangement (Fig. 1B). Chronically injured tendons were also enlarged compared to normal and exhibited a thickened fibrosed paratenon (Fig. 1C). By this stage granulation tissue was absent but the highly organised fascicular arrangement present in normal tendon was not restored. Histology of normal equine SDFT's showed a highly organised and regular arrangement of parallel collagen fibrils with tenocytes (tendon fibroblasts) arranged along and between the fibrils (Fig. 1D). In contrast, sub-acutely injured tendons exhibited neovascularisation and fibroplasia, with disrupted collagen fibril organisation and marked increased cellular infiltration (Fig. 1E). Chronically injured tendons (Fig. 1F) displayed more regular arrangement of collagen fibrils, with well established neo-vascularisation, reactive fibroplasia and increased cellular infiltration with Mϕ localised to peri-vascular and endotenon regions (Fig. 2A–C). In chronic injury, Mϕ were also located at the interface between the site of previous injury and the adjacent more normal tendon (Fig. 2D).

### Double immunostaining of SDFT reveals a shift in macrophage polarity

**Figure 3:**
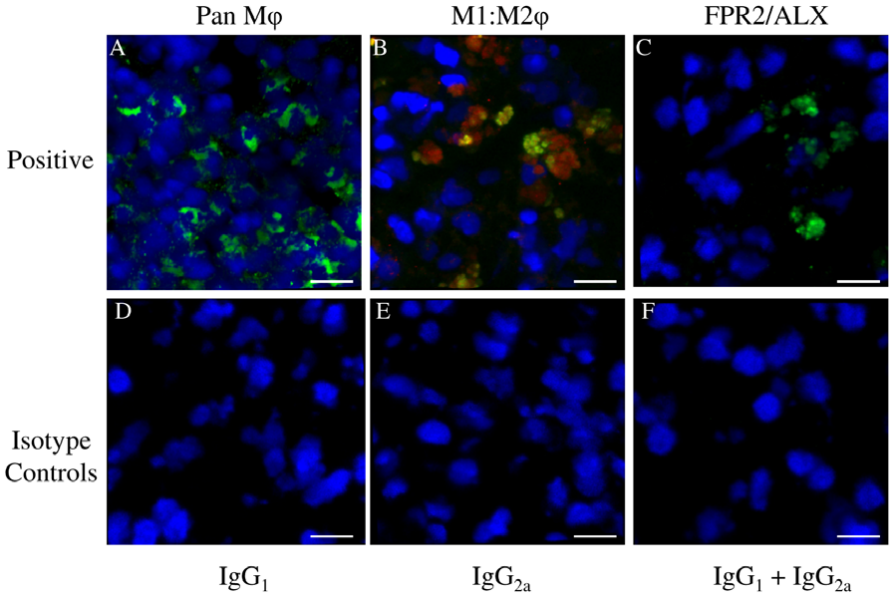
Representative Z stack images of antibody control cryosections of equine spleen. Panel A–C represents positive controls for: (A)CD172a Mϕ (green); (B) Dual antibody labelling with CD14 (M1Mϕ red) and CD206 (M2Mϕ green) showing co-expression of both markers (CD14^high^CD206^high^) in splenic monocytes/macrophages; (C) Lipoxin A_4_ receptor (FPR2/ALX, green). Panel D–F represents negative controls, consisting of murine isotype matched primary control antibodies: (D) IgG_1_, (E) IgG_2a_, (F) IgG_1_ and IgG_2a_. Blue represents Hoechst nuclear counter stain. Scale bar = 12 µm.

**Figure 4:**
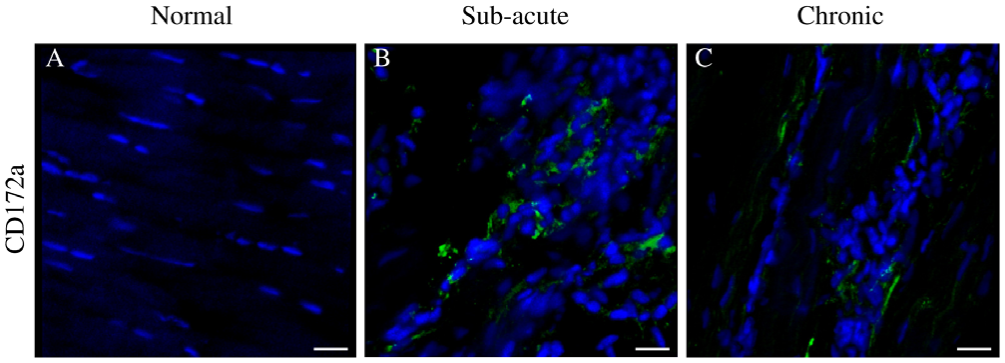
Panel of representative 3-dimensional reconstructed Z stack immunofluorescent low magnification images of equine SDFT sections. Pan Mϕ (CD172a) staining is shown for is for (A) normal, (B) sub-acute and (C) chronic injured tendons. Immunopositive cells are green; blue represents Hoechst nuclear counter stain. Scale bar = 20 µm.

**Figure 5:**
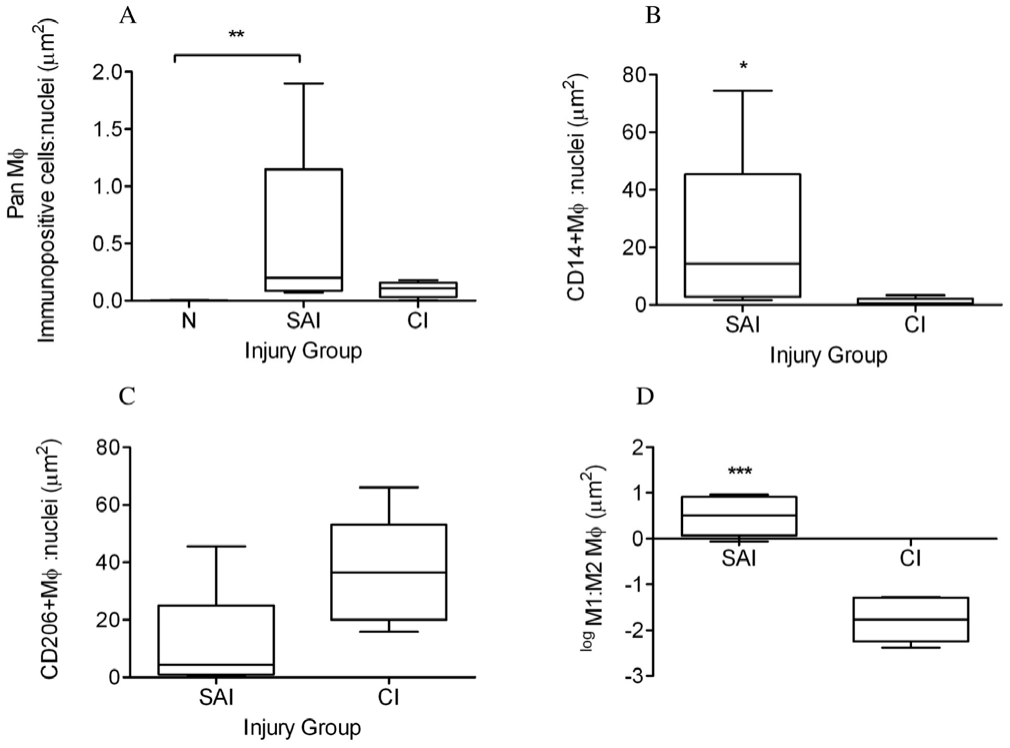
Box plots illustrating ratios of areas (µm^2^) of immunopositive cells: counterstained nuclei. (A) CD172a pan Mϕ in normal (uninjured), sub-acute and chronic injured equine tendons; (B) CD14^high^ (M1Mϕ) and (C) CD206^high^ (M2Mϕ) expression in sub-acute and chronic injured tendons respectively. (D) Log transformed ratio of areas of positive immuno-reactivity for M1∶M2 Mϕ from dual labelled CD14 and CD206 sections of sub-acute and chronic injured tendons. SAI = sub-acute injury (n = 5), CI = chronic injury (n = 5), N = normal tendon (n = 5). All values represent median with maximum and minimum range. *** *P*<0.001, ** *P*<0.01, * *P*<0.05.

**Figure 6:**
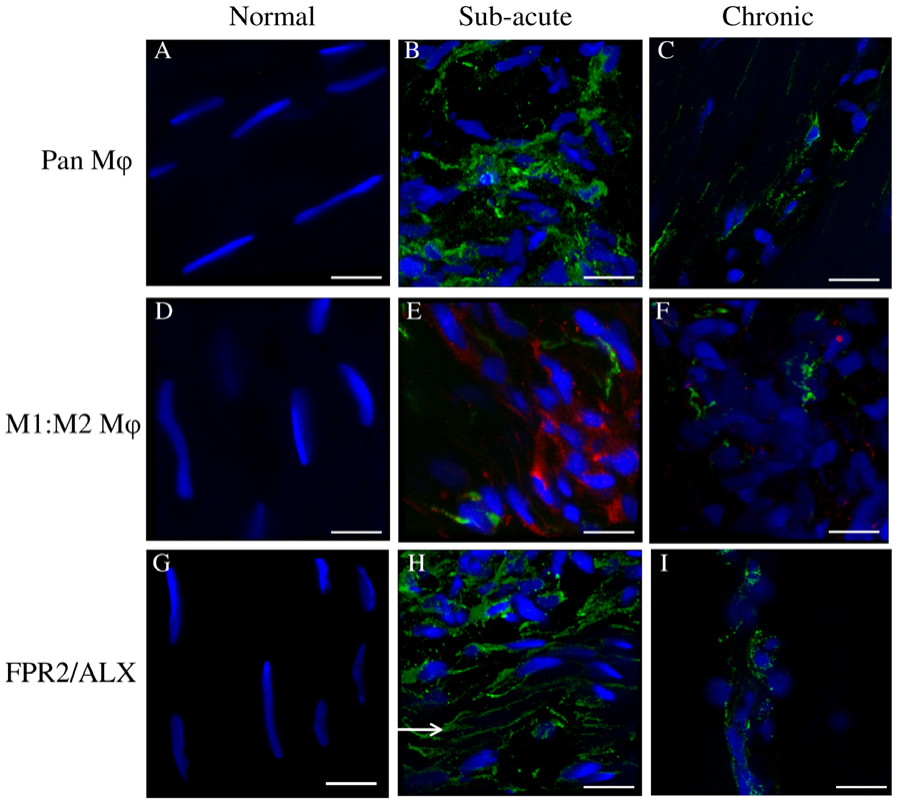
Panel of representative 3-dimensional reconstructed Z stack immunofluorescent images of equine SDFT sections. (A–C) CD172a Mϕ antibody, immunopositive cells are green; (D–F) Dual antibody labelling for CD14 (M1Mϕ red) and CD206 (M2Mϕ green) showing CD14^high^CD206^low^ Mϕ in sub-acute injury (E) and CD14^low^CD206^high^ Mϕ in chronic injury (F); (G–I) FPR2/ALX, immunopositive cells are green. Arrow in panel H shows FPR2/ALX expression on tenocyte cytoplasmic extensions. Panels A, D, & G are from normal uninjured tendon; B, E & H are from sub-acutely injured tendon; C, F & I are from chronically injured tendon. Blue represents Hoechst nuclear counter stain. Scale bar = 12 µm.

**Figure 7:**
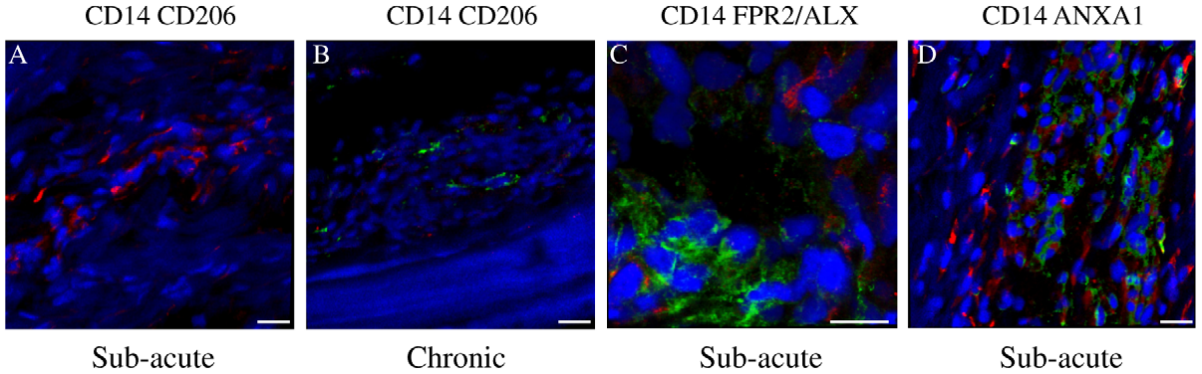
Panel of representative 3-dimensional reconstructed Z stack immunofluorescent images of injured SDFT sections. (A) Dual labelled CD14 (red) and CD206 (green) showing predominance of CD14^high^ Mϕ in sub-acute tendon injury. Scale bar = 20 µm. (B) Dual labelled CD14 (red) and CD206 (green) showing predominance of CD206^high^ Mϕ in chronic tendon injury. Scale bar = 20 µm. (C) Dual labelled CD14 (red) and FPR2/ALX (green), showing FPR2/ALX expression by tenocytes but not M1Mϕ in sub-acute tendon injury. Scale bar = 12 µm. (D) Dual labelled CD14 (red) and Annexin A1 (green), showing Annexin A1 expression by tenocytes but not M1Mϕ in sub-acute tendon injury. Scale bar = 20 µm. Blue represents Hoechst nuclear counter stain.

Images for the positive, negative and isotype controls for all antibodies were validated on cryosections of equine spleen and are shown in Fig. 3 A–F. Co-expression of CD14 and CD206 (demonstrated by yellow staining) was not identified in sub-acute or chronic injured tendons in contrast to equine spleen (Fig. 3 B). Immunostaining for the pan Mϕ marker CD172a on cryosections derived from normal, sub-acute and chronic injured SDFT's (Fig.4 A–C) revealed a greater number of Mϕ in sub-acutely injured SDFT compared to normal SDFT (*P* = 0.008), with fewer Mϕ present in chronic injured tendons (Fig. 5A). Dual labelling using antibodies to CD14 and CD206 facilitated characterisation of Mϕ sub-populations through the stages of tendon healing (Fig. 6 D–F). There was a significantly greater proportion of M1Mϕ phenotype (CD14^high^CD206^low^) in sub-acute injury compared to chronic injured SDFT (*P* = 0.01) (Figs. 5B, 6E and 7A). In contrast, the M2Mϕ phenotype (CD14^low^CD206^high^) was predominant during the chronic injury phase (Figs. 5C, 6F and 7B); these cells were located in the peri-vascular and endotenon regions. To facilitate direct comparison of Mϕ phenotypes during sub-acute and chronic injury phases, a ratio of areas (µm^2^) of positive immuno-reactivity for M1∶M2 Mϕ was derived. This showed predominance of the M1 polarised Mϕ phenotype in sub-acute injury compared to chronic injury (*P*<0.001) (Fig. 5D).

### Expression of FPR2/ALX is upregulated during the sub-acute phase of tendon injury

**Figure 8:**
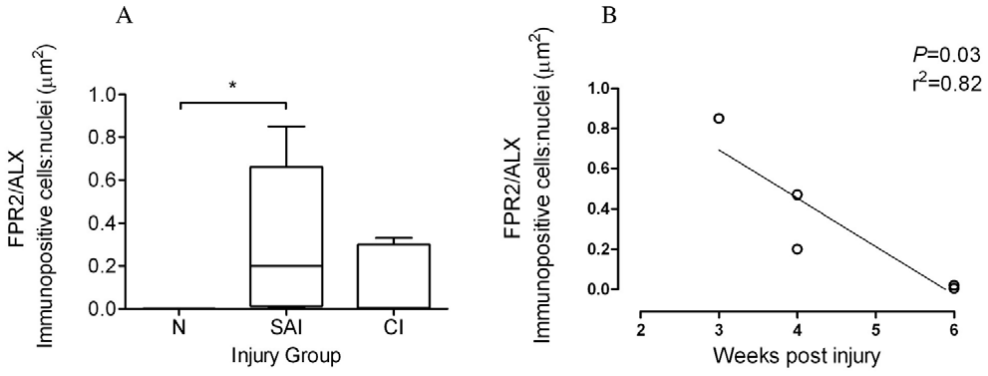
FPR2/ALX expression in normal and injured equine tendons. (A) Box plot illustrating ratio of areas (µm^2^) of immunopositive cells: counterstained nuclei of expression of the Lipoxin A_4_ receptor (FPR2/ALX) in normal (N, n = 5), sub-acute (SAI, n = 5) and chronic injured (CI n = 5) equine tendons. Values represent median with maximum and minimum range. * *P*<0.05. (B) FPR2/ALX expression in sub-acutely injured tendons (n = 5) showing significant decline in FPR2/ALX protein expression with time after injury (*P* = 0.03, r^2^ = 0.82).

To assess whether changes in Mϕ subsets are accompanied by changes in FPR2/ALX expression, we next analysed expression of this protein in the same normal, sub-acute and chronic injured SDFT samples (Fig. 6 G–I). There was significantly greater FPR2/ALX expression in sub-acute but not chronic injured SDFT compared to normal tendons (*P* = 0.01) (Fig. 8A). When separated into the early and late stages of sub-acute injury, there was a significant decline in FPR2/ALX expression with increasing time after injury (*P* = 0.03, r^2^ = 0.82 Fig. 8B), which may account for the large range of FPR2/ALX expression across this group. FPR2/ALX was expressed on cytoplasmic processes of tenocytes of sub-acutely injured tendons (Fig. 6H) but not by M1Mϕ, as verified by dual labelling for CD14 and FPR2/ALX on tendon sections (Fig. 7C).

### Effect of pro-inflammatory mediators on FPR2/ALX expression

**Figure 9:**
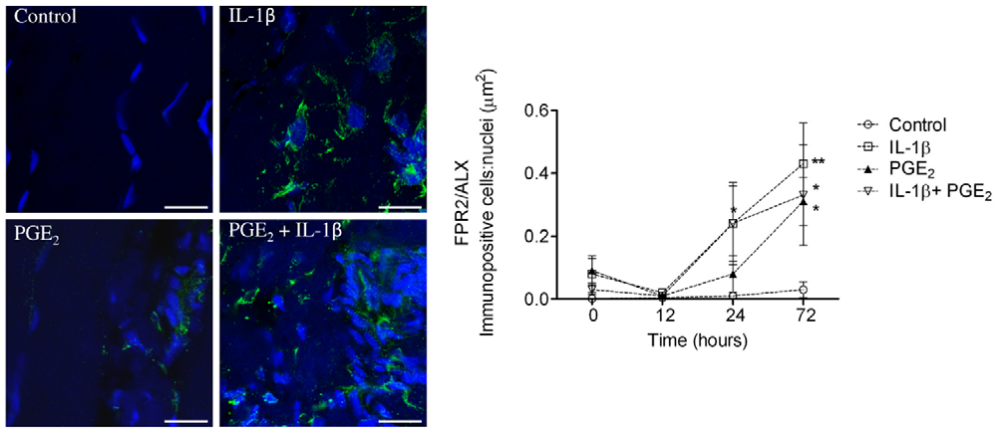
Panel of representative 2-dimensional images illustrating FPR2/ALX expression in tendon explants showing non-stimulated (vehicle only) control compared to explants stimulated with 5 ng ml^−1^ IL-1β and 1.0 µM PGE_2_ either alone or in combination. Immunopositive staining is green, with Hoechst nuclear counter stain in blue. Scale bar = 12 µm. Graph showing the effect of pro-inflammatory mediators on tendon FPR2/ALX expression *in vitro*. Data represent average FPR2/ALX expression in tendon explants derived from 3 normal (uninjured) horses, whereby 2 replicates were analysed for each experimental condition and time point per horse. FPR2/ALX expression was determined at time points 0, 12, 24 and 72 hours after stimulation and compared to non-stimulated controls. Significant up-regulation of FPR2/ALX occurs 24 hours after stimulation with IL-1β or a combination of both mediators, and maximally 72 hours after stimulation with IL-1β, PGE_2_ or combination of both mediators compared to controls. ** *P*<0.01, * *P*<0.05. Error bars denote SEM.

As FPR2/ALX was absent in normal SDFT, but present during sub-acute and chronic injury, we next assessed whether FPR2/ALX expression was influenced by the presence of pro-inflammatory mediators that are known to be upregulated in tendon injury. We used an *in vitro* tendon explant model, whereby macroscopically normal explants were stimulated with IL-1β and PGE_2_, either alone or in combination, and analysed FPR2/ALX expression over time. Representative images for FPR2/ALX expression 72 hours post stimulation and average data for tendon explants from the 3 horses are shown in Fig. 9. There was a significant effect of time (*P*<0.001) and experimental condition (*P* = 0.01) on FPR2/ALX expression. FPR2/ALX expression was at baseline or low level between 0–72 hours in non-stimulated control tendon explants. Stimulation with IL-1β alone or in combination with PGE_2_ significantly increased FPR2/ALX expression by tenocytes compared to non-stimulated controls at 24 hours (*P* = 0.049 and *P* = 0.02 respectively). In contrast there was no significant effect of PGE_2_ in isolation at this time point. Maximal FPR2/ALX expression occurred 72 hours after stimulation with either IL-1β or PGE_2_ compared to controls (*P* = 0.002 and *P* = 0.01 respectively). Combined stimulation with both mediators also resulted in increased FPR2/ALX expression compared to controls at 72 hours (*P* = 0.02); however this was not significantly different to expression observed with stimulating with either mediator alone.

### Effect of pro-inflammatory mediators on lipoxin A_4_ release in media

**Figure 10:**
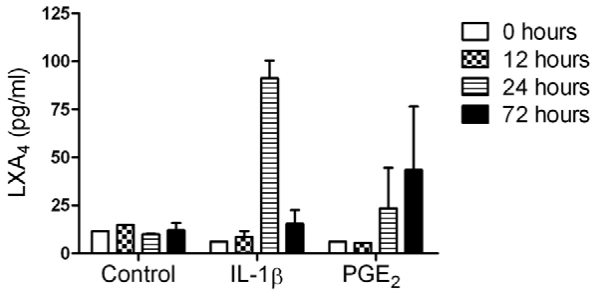
The effect of pro-inflammatory mediators on release of lipoxin A_4_ (LXA_4_) in media from tendon explants. Data represent average LXA_4_ levels from 3 normal (uninjured) horses. LXA_4_ levels were determined at time points 0, 12, 24 and 72 hours after stimulation and compared to non-stimulated controls. There was a difference in the temporal response showing increased LXA_4_ release 24 hours after stimulation with IL-1β (*P*<0.001) and 72 hours after stimulation with PGE_2_ (*P* = 0.048) compared to non-stimulated controls. Values for significance were based on a linear model.

In addition to assessing the effect of pro-inflammatory mediators on FPR2/ALX expression in tendon explants *in vitro*, we also investigated their effect upon the release of LXA_4_ into tissue culture media. LXA_4_ production was at baseline or low level (<15 pg/ml) between 0–72 hours in media from non-stimulated control explants. Stimulation with IL-1β induced maximal LXA_4_ release 24 hours post stimulation compared to controls (6-fold increase, *P*<0.001) but returned to baseline levels at 72 hours. In contrast, LXA_4_ levels increased maximally at 72 hours after PGE_2_ stimulation compared to controls (3-fold increase, *P* = 0.048) (Fig. 10).

### Expression of Annexin A1 is upregulated during tendon injury

**Figure 11:**
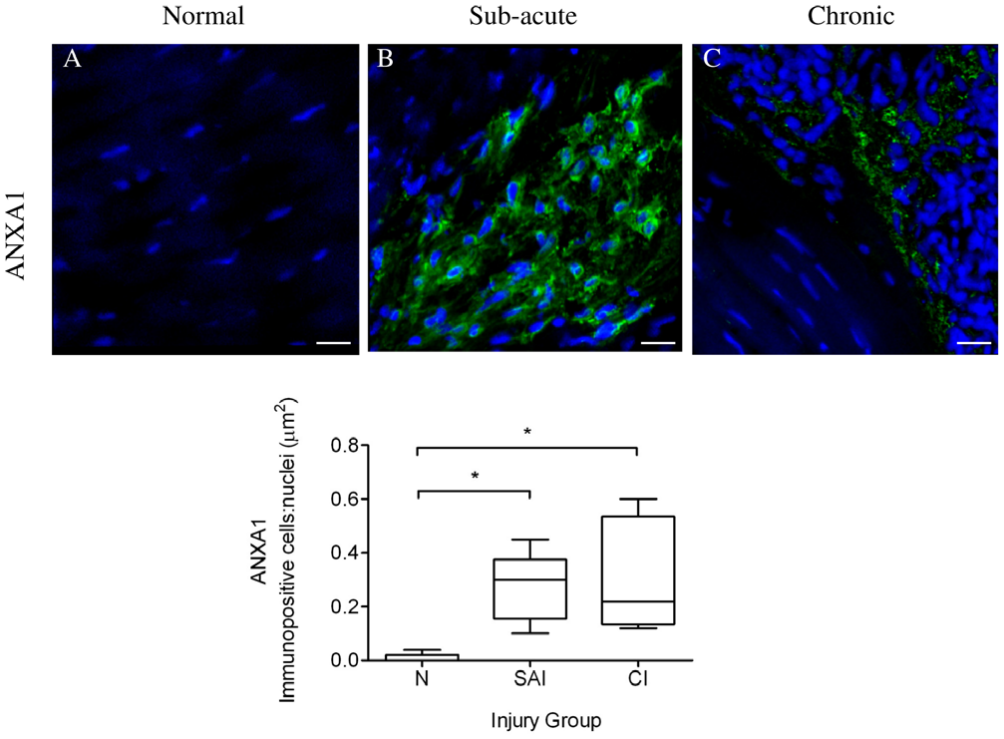
Panel of representative 3-dimensional reconstructed Z stack immunofluorescent images of equine SDFT sections. Annexin A1 (ANXA1) staining is shown for is for (A) normal, (B) sub-acute and (C) chronic injured tendons. Immunopositive cells are green; blue represents Hoechst nuclear counter stain. Scale bar = 20 µm. Box plot shows significantly increased Annexin A1 expression in sub-acute and chronic injured tendons compared to low level expression in normal tendons. SAI = sub-acute injury (n = 5), CI = chronic injury (n = 5), N = normal tendon (n = 5). Values represent median with maximum and minimum range. * *P*<0.05.

Annexin A1 is also a ligand for FPR2/ALX with additional roles in inflammation, resolution and apoptosis. To assess expression of Annexin A1 in tendon injuries, we next analysed expression of this protein in the same normal, sub-acute and chronic injured SDFT samples. Annexin A1 expression was restricted to tenocytes in injured tendons and not M1Mϕ, as co-expression of these markers was not observed (Fig. 7D). Annexin A1 expression was significantly increased in both sub-acute and chronic injured tendons compared to normal samples, which showed very low level expression (*P*<0.01) (Fig. 11).

## Discussion

In the present study, we describe changes in Mϕ subsets, FPR2/ALX and Annexin A1 expression in the sub-acute and chronic phases of equine flexor tendon injuries. Our study illustrates that the number of Mϕ significantly increase in sub-acutely injured equine flexor tendons (3–6 weeks post injury) in contrast to their absence in normal uninjured tendon. When the injury progressed from sub-acute to the chronic injured stage, there was a potential shift from a pro-inflammatory M1Mϕ (CD14^high^CD206^low^) to an anti-inflammatory M2Mϕ (CD14^low^CD206^high^) phenotype. Furthermore, tendon injury led to an up-regulation of FPR2/ALX in tenocytes in the sub-acute injury stage, which is likely to be mediated by the production of pro-inflammatory mediators by either tenocytes or infiltrating Mϕ. Annexin A1 expression was also significantly increased in sub-acute and chronic tendon injuries compared to normal tendons. Our study provides novel data illustrating components of inflammation are highly active during both the early and chronic stages of naturally occurring equine flexor tendon injury, which have not been described during the early phase of equivalent injury in man. Similarly, it is not possible to recapitulate chronic injury or re-injury using induced models of murine tendon injury, and may be more appropriate from a therapeutic perspective.

The crucial role of Mϕ as both effectors of tissue injury and repair are well documented in other healing connective tissues [65], [66]. Mϕ are responsible for executing specific functions during the diverse phases of wound repair as suggested by sub-optimal wound healing in Mϕ deficient injured sites [67] and murine knockout models [15], [68]. Deficiency of Mϕ, particularly during the early stage of repair has profound adverse effects on healing, including reduced granulation tissue formation and impaired epithilialization [16]. The plasticity and functional polarisation of tissue Mϕ that we observed in injured tendon was similar to that reported in skin repair [16]. Fully polarised M1 and M2 Mϕ have been described as the extreme phenotypes of a continual spectrum of functional states [28]. The greater proportion of M1Mϕ polarised phenotypes (CD14^high^CD206^low^) in sub-acute compared to chronic injury was indicative of the highly pro-inflammatory status of recent injury in the sub-acutely injured tendon samples. The predominance of M2Mϕ (CD14^low^CD206^high^) in chronic tendon injury suggests a potential phenotype or population change. Although we have not investigated the nature of the stimulus governing the fate of the M1Mϕ, this has been suggested to occur by either apoptosis or lymphatic clearance [69]. We observed M2Mϕ in peri-vascular and endotenon regions, and at the interface between the previously injured area and adjacent more normal tendon, a site which is prone to re-injury due to disparate tissue biomechanical properties at this interface [11]. We hypothesise that M2Mϕ in this region are actively surveying the healing tendon for ‘stress signals’ in order to protect this susceptible region from re-injury. It is likely that cyclical loading sustains an inflammatory response that M2Mϕ fail to adequately resolve, resulting in the development and accumulation of localised collagen fibril damage (micro-lesions) during exercise rehabilitation. This innate failure to resolve inflammation may increase the propensity for fibrotic repair of adult connective tissues in contrast to the abilities of macrophage deficient foetal wounds that heal with minimal scar formation [16], [36]. Thus the persistence of Mϕ appears to be correlated with the deposition of scar tissue at the site of injury in tendons. We did not observe co-expression of CD14 and CD206 in the injured tendon sections analysed. This may reflect the presence of specific sub-sets of differentiated tissue Mϕ in tendon, because co-expression was observed in equine spleen in the mixed population of circulating monocytes as previously described [30], [31].

FPR2/ALX is reported to have a central role in controlling the duration and magnitude of the inflammatory response [44], [70]. Although FPR2/ALX expression has not previously been described in tendon, our study shows significant upregulation of FPR2/ALX expression on tenocytes in the sub-acute phase of injury, with lower level expression in chronic injury and not detectable in normal (uninjured) tendon. The binding of LXA_4_ to FPR2/ALX on tenocytes may enhance paracrine related reprogramming of cytokine primed M1Mϕ [71], facilitating the potential phenotypic switch we observed during tendon healing. Indeed, endogenous LXA_4_ circuits are cited as regulators of inflammatory angiogenesis and neovascularisation [72], both of which are also prominent clinical features of early stage equine tendinopathy. FPR2/ALX has been reported to be expressed on circulating monocytes and Mϕ [40], but we were unable to show expression on M1Mϕ in dual labelled sections of sub-acutely injured tendons. The apparent absence of FPR2/ALX expression on M1Mϕ may be attributable to phenotypic changes that occur in the transition from infiltrating monocytes at injured sites to differentiated tissue Mϕ [73]. We speculate diminished FPR2/ALX expression by Mϕ may contribute to the defective healing that occurs in injured tendons.

We also identified tenocytes expressing Annexin A1 (an additional ligand for FPR2/ALX) in sub-acute and chronic injured tendons. Annexin A1 has not been previously described in tendon; however it is reported to act as an endogenous anti-inflammatory mediator complimenting the actions of lipoxins, potentiating resolution of inflammation via FPR2/ALX [74]. Furthermore, as Annexin A1 is also implicated in promoting pro-apoptotic mechanisms [75], its presence in tendon may have an additional role in the identification of damaged tenocytes that are to be removed by Mϕ after injury. As Annexin A1 protein was significantly upregulated in both sub-acute and chronic phases of tendon injury, it would appear that inflammation and apoptosis continue for a prolonged period after initial injury and throughout tendon healing and remodelling.

Regulation of FPR2/ALX by pro-inflammatory mediators has not been previously reported in wound healing, although transcription of FPR2/ALX was shown to be upregulated by IL-13, IL-4, IL-6 and IL-1β in human enterocytes [76]. In our study, stimulation of normal tendon explants with IL-1β or PGE_2_ induced maximal release of LXA_4_ at 24 and 72 hours respectively, compared to non-stimulated controls. The differences in kinetics seen are potentially attributed to differences in receptor involvement and subsequent down-stream signalling between IL-1β and PGE_2_ [77], [78], [79], or the class switching of arachadonic acid derived eicosanoids from prostaglandins towards production of LXA_4_ [52]. Alternatively, these data may suggest that IL-1β is a more potent activator of LXA_4_ than PGE_2_, as peak LXA_4_ release coincided with the observed upregulation of FPR2/ALX expression at 24 hours and maximally at 72 hours after stimulation with IL-1β. Interestingly, combined stimulation with both mediators did not significantly increase FPR2/ALX expression compared to using either mediator in isolation. The lack of an additive effect of the two pro-inflammatory mediators may suggest maximal FPR2/ALX transcription level is attained by stimulation with either mediator alone, and we speculate that this may be a mechanism to tightly regulate this gene.

M1Mϕ are reported to secrete pro-inflammatory agents such as IL-1β and PGE_2_ [18], [28]. In support of our *in vitro* experimental findings, the presence of M1Mϕ also coincided with FPR2/ALX up-regulation in naturally occurring early stage tendon injury. Hence, FPR2/ALX may be acting in an endogenous protective capacity to limit the degree of damage to the extracellular matrix of recently injured tendon. However the decline in FPR2/ALX expression in the late sub-acute stage of tendon injury suggests it is possible that the duration and magnitude of FPR2/ALX expression are insufficient to promote complete resolution of inflammation in injured tendon, which may contribute to the development of chronic inflammation and fibrotic repair [46], [69], [71]. As M2Mϕ in chronic injury produce IL-1RA [28], reduced FPR2/ALX expression in chronic compared to sub-acute injury may be attributable to the reduced IL-1β effects on tendon. Improved understanding of inflammation is crucial to the development of novel anti-inflammatory agents that both preserve the beneficial, yet enhance removal of the detrimental components of the cascade. It is possible that augmenting resolution by ‘tricking’ chronically inflamed tissues into a resolving pathway using potential new therapeutic targets such as lipoxins and other FPR2/ALX agonists [46], [80], [81] may reduce the propensities for fibrotic tendon repair and re-injury.

The categorisation of injury stage in this study was based on known clinical history and post mortem examination, which may not account for the presence of sub-clinical inflammatory processes that occur prior to the onset of clinical disease, either in older animals or those subjected to higher levels of exercise. We believe that our present study contributes to the evidence that whilst symptomatic inflammation is only subtle and transient in tendinopathies, the regulatory components of the inflammatory response in early stage tendinopathy are in fact highly active at the cellular level. Consequently, prolonged subclinical inflammation can potentially develop into symptomatic injury if sufficient extracellular matrix microdamage accumulates. The ability to determine the nature of the Mϕ phenotype and FPR2/ALX status of sub-clinically injured tendon would provide valuable insight to the inflammatory processes occurring prior to the onset of clinical disease; however this is currently precluded by our inability to accurately identify sub-clinical injuries *in vivo*. Future research should aim to identify parameters that determine the inflammatory and resolution status of injured tendon *in vivo*, such that sub-clinical injury can be identified and treated prior to onset of clinical disease.

## Materials and Methods

### Ethics Statement

Ethics approval for the collection of post mortem equine tendons from an abattoir or local equine veterinary referral hospital for this study was sought and approved from the Ethics and Welfare Committee at the Royal Veterinary College (URN 2011 1117). No horses were euthanased for obtaining tissues for the purposes of this study and all tendons were harvested after informed consent had been obtained.

### Collection and processing of equine tendons

Equine forelimbs (normal, without tendon injury; n = 5, mean age 8±3.5 years) were obtained from a UK abattoir and the tensile region of the SDFT was dissected within 4 hours of death. Tendons were classified as normal based on their macroscopic post mortem appearance which included lack of visible signs of swelling of the tendon body and a consistent pattern of fascicles on transverse sections. Sub-acute (history of injury 3–6 weeks old; n = 5, mean age 10±4.8 years) and chronically injured tendons (>3 months post injury; n = 5, mean age 13±4 years) were sourced from either an abattoir or a local equine veterinary referral hospital and similarly processed. Tendon injuries were aged based on historical information obtained from either the owner or referring veterinary surgeon prior to euthanasia of the horse. All harvested tendons were obtained from Thoroughbred or Thoroughbred cross breed horses aged between 4 and 16 years. Tendon pieces from the mid-tensional regions were embedded in optimal cutting temperature compound (OCT, Sakura Tissue-Tek®, Alphen aan den Rijn, The Netherlands) and snap frozen in pre-chilled (−80°C) n-hexane and stored at −80°C until processed for histological or immunofluorescent analysis. Serial sections (8–10 µm thickness) were cut on a cryostat (Bright, Cambridge, UK) and mounted onto poly-L-lysine coated slides (VWR, Lutterworth, UK) and allowed to dry for 2 hours at room temperature prior to storage at −80°C.

### Haematoxylin & Eosin staining of tendon cryosections

Tendon cryosections were fixed in chilled (4°C) acetone for 5 minutes and then allowed to air dry for 30 minutes. Sections were hydrated in Phosphate Buffered saline (PBS) and stained with Gills III Haematoxylin (Sigma Aldrich, Dorset, UK) for 3 minutes and then washed with running water for a further 3 minutes. Slides were stained with alcoholic Eosin (Sigma Aldrich,) for 40 seconds and rinsed with water. After dehydration and a final rinse using Histosol™ histological clearing agent (National Diagnostics, Hull, UK), slides were mounted using DPX mounting medium (BDH, Poole, UK).

### Detection and characterization of macrophages, FPR2/ALX and Annexin A1 in normal and injured equine tendons

Cryosections of equine spleen were used as positive control tissue to validate suitability and dilution of antibodies for use on tendon sections. Negative controls consisted of spleen and sub-acutely injured SDFT cryosections incubated with murine isotype matched primary control antibodies (Southern Biotech, Birmingham, AL, USA). Subsequently, consecutive cryosections of normal and injured equine tendons were probed using mouse anti-human monoclonal antibodies to CD172a (pan Mϕ marker, IgG_1_, VMRD, Pullman WA, USA), CD14 (M1 polarised Mϕ phenotype, IgG_2a_, BioLegend, Cambridge, UK), CD206 (M2 polarised Mϕ phenotype, IgG_1_, AbCam, Cambridge, UK), FPR2/ALX (Lipoxin A_4_ receptor, IgG_1_, AbCam, Cambridge) and Annexin A1 (IgG_1_, Abcam, Cambridge). Although CD172a is also a granulocyte marker, we verified that granulocytes were not present using Haematoxylin & Eosin stained cryosections of sub-acute and chronic injured tendons. After warming cryosections at room temperature for 30 minutes, sections were rehydrated in 1% PBS-Tween 20 (PBS-T) and blocked in PBS containing 5% normal goat serum (Sigma Aldrich) for 1 hour in a humid chamber at room temperature. This was followed by incubation with the primary antibody in PBS containing 5% normal goat serum for 2 hours, diluted either 1∶100 or 1∶50 (CD206). After 3×5 minute washes in PBS-T, sections were incubated with the respective goat anti-mouse secondary antibodies conjugated with either AlexaFluor® 488 IgG, Alexa Fluor® 594 IgG_2a_ (Invitrogen, Paisley, UK), or goat anti-mouse IgG_1_-R-PE (Southern Biotech), each diluted 1∶300 in PBS containing 5% normal equine serum (Sigma Aldrich) for 2 hours. To visualise the nuclei, slides were subsequently counterstained with 0.5 µgml^−1^ Hoechst 33342 (Invitrogen) for 20 minutes and washed in PBS-T. To quench tissue fluorescence, slides were incubated with 0.1% Sudan Black B (BDH, Poole, UK) in 70% ethanol for 20 minutes [82], washed in PBS-T and mounted using a solution of 80% glycerol in 0.5 mM Tris buffer (pH 7.2). Slides were stored at 4°C until image acquisition. In order to identify the predominating Mϕ populations in sub-acute and chronically injured tendon, dual staining using antibodies against CD14 and CD206 was performed. Due to the lack of commercially available antibodies to Lipoxin A_4_ for immunofluorescence, Annexin A1 was chosen as an alternative ligand for FPR2/ALX. Dual staining was also performed using antibodies against CD14 and FPR2/ALX or CD14 and Annexin A1 to determine expression on tenocytes and M1Mϕ.

### Effect of pro-inflammatory mediators on FPR2/ALX expression

As evaluation of injured tendon sections revealed differences in FPR2/ALX expression, we assessed the impact of pro-inflammatory mediators on FPR2/ALX expression *in vitro*. Macroscopically normal SDFTs from an equine abattoir were harvested from 3 Thoroughbred horses aged between 2 and 15 years (mean age 8 years). Tendons were cut into 0.4 cm^3^ explants (300 mg±30 mg) under aseptic conditions and incubated at 37°C and 5% CO_2_ under humidified atmosphere in 3 ml Dulbecco's Modified Eagle Medium (PAA, Somerset, UK) containing 1% Penicillin and Streptomycin (PAA) without foetal calf serum. Samples were stimulated with either 5 ng ml^−1^ human recombinant IL-1β (Merck, Calbiochem®, Nottingham UK), 1.0 µM PGE_2_ (Sigma Aldrich) or a combination of 5 ng ml^−1^ IL-1β and 1.0 µM PGE_2_. Non-stimulated (vehicle only) samples served as controls. Tendon explants and media were harvested immediately (time 0) and 12, 24 and 72 hours after stimulation. Media samples were stored at −80°C prior to processing for determination of LXA_4_ levels. Tendon explants were snap frozen in chilled (−80°C) n-hexane, embedded in OCT and stored at −80°C until processing for immunofluorescent analysis. Cryosections were cut and mounted onto poly-L-lysine coated slides as described above. Sections were allowed to dry for 2 hours at room temperature prior to storage at −80°C. Cryosections were probed with a 1∶100 diluted primary mouse monoclonal FPR2/ALX antibody (Lipoxin A_4_ receptor, IgG_1_, AbCam) and secondary goat anti-mouse IgG_1_ (Southern Biotech), followed by Hoechst nuclear counter stain. Tissue fluorescence was quenched with Sudan Black B (BDH) and sections were mounted for imaging as before.

### Image acquisition and analysis

Images for histology sections were acquired using a Leica DM4000B microscope with a DC500 camera (Leica Microsystems, Milton Keynes, UK). All immunofluorescent images were recorded on a Leica SP5 confocal microscope (Leica Microsystems) using a 63× oil immersion objective (NA = 1.4). For 2-dimensional images a minimum of 5 random high power field images were acquired per tissue section per horse (for analysis of normal and naturally diseased tendon), or per experimental condition for each time point (*in vitro* explant experimental system). Antigen expression was automatically quantified by measurement of the area of immunopositive cells in each image. As we expected this area to vary with the cellular density in the tendon, the area of immunopositive cells was standardised by expressing it as a ratio to the total area of counterstained nuclei, which was used as a measure of cellular density. Quantitative analysis was performed on 2-dimensional images which were acquired using a 63× oil immersion objective for pan Mϕ, FPR2/ALX and Annexin A1 antibody treated cryosections. In order to determine the predominant Mϕ populations by dual antibody labelling, a ratio of areas (µm^2^) of positive immuno-reactivity for CD14 (red) versus CD206 (green) was determined from 2-dimensional images acquired from 5 horses each with either sub-acute or chronic tendon injury. In each case, the area of positive staining was determined automatically using Volocity software (PerkinElmer, Cambridge, UK). Positive staining was defined as a signal being over a predetermined threshold for each stain. Groups of less than 4 continuous positive pixels were considered noise and excluded from the measurements. Z stack images were acquired using either 20× or 63× objectives with 256×256 matrix and slice thickness between 0.18–0.25 µm. Three-dimensional image reconstructions were created using Volocity software (PerkinElmer).

### Measurement of lipoxin A_4_ in media from stimulated tendon explants

In addition to assessing FPR2/ALX expression in tendon explants stimulated with IL-1β or PGE_2_, media were analysed to determine release of the ligand LXA_4_. LXA_4_ was separated from the supernatant by passage through Bond Elut C18 columns (Agilent Technologies, CA, USA) followed by elution with methyl formate. Samples were evaporated to dryness in a stream of nitrogen and resuspended in extraction buffer from a LXA_4_ ELISA kit (Neogen Corp, Lexington, KY, USA). LXA_4_ levels in media samples were determined using this ELISA in duplicate wells according to the manufacturer's instructions. The ELISA kit is specific for LXA_4_ showing minimal cross-reactivity [LXA_4_ 100%, Lipoxin B_4_ 1.0%, 15-hydroxyeicosatetraenoic acid (HETE) 0.1%, 5-HETE <0.1% and 12 HETE <0.1%].

### Statistics

For normal and injured tendons, statistical analyses were performed on 2-dimensional confocal images using GraphPad Prism 5 (GraphPad Software Inc., San Diego, CA). Normality was tested using a Kolmogorov-Smirnov test. Kruskal-Wallis with post hoc Dunn's multiple comparison tests were performed to determine differences between normal, sub-acute and chronic injured tendons for CD172a (pan) Mϕ, FPR2/ALX and Annexin A1 staining. Mann Whitney tests were used to compare expression of CD14^high^CD206^low^ (M1Mϕ) and CD14^low^CD206^high^ (M2Mϕ) in sub-acute and chronic injured tendons. To determine the predominating Mϕ phenotype in both sub-acute and chronic injury stages, a Student's t test was performed on log transformed ratios of areas of positive immuno-reactivity for M1∶M2 Mϕ. In all cases, the *P* value was considered significant if below 0.05.

For FPR2/ALX explant experiments, statistical analyses for explant experiments were performed using SPSS PASW Statistics 18 (SPSS Inc Illinois, USA). A linear mixed effect model was used to assess FPR2/ALX expression on natural log transformed data to account for repeat measures of experimental condition and time points 0, 12, 24 and 72 hours. A linear model was used to assess LXA_4_ levels in media to account for effects of horse, experimental condition, time and interaction between condition and time. Linear contrast was then used on each data set to assess the differences between each experimental condition and time point. *P*<0.05 was considered statistically significant.
